# Safety Implications of Modulating Nuclear Receptors: A Comprehensive Analysis from Non-Clinical and Clinical Perspectives

**DOI:** 10.3390/ph17070875

**Published:** 2024-07-03

**Authors:** Mohan Rao, Eric McDuffie, Sanjay Srivastava, Warren Plaisted, Clifford Sachs

**Affiliations:** 1 Toxicology Department, Neurocrine Biosciences, Inc., San Diego, CA 92130, USAcsachs@neurocrine.com (C.S.); 2 Chemistry Department, Neurocrine Biosciences, Inc., San Diego, CA 92130, USA; 3 Biology Department, Neurocrine Biosciences, Inc., San Diego, CA 92130, USA

**Keywords:** toxicoinformatics, big data analytics, nuclear receptor modulation, gene expression analysis, off-target interactions

## Abstract

The unintended modulation of nuclear receptor (NR) activity by drugs can lead to toxicities amongst the endocrine, gastrointestinal, hepatic cardiovascular, and central nervous systems. While secondary pharmacology screening assays include NRs, safety risks due to unintended interactions of small molecule drugs with NRs remain poorly understood. To identify potential nonclinical and clinical safety effects resulting from functional interactions with 44 of the 48 human-expressed NRs, we conducted a systematic narrative review of the scientific literature, tissue expression data, and used curated databases (OFF-X™) (Off-X, Clarivate) to organize reported toxicities linked to the functional modulation of NRs in a tabular and machine-readable format. The top five NRs associated with the highest number of safety alerts from peer-reviewed journals, regulatory agency communications, congresses/conferences, clinical trial registries, and company communications were the Glucocorticoid Receptor (GR, 18,328), Androgen Receptor (AR, 18,219), Estrogen Receptor (ER, 12,028), Retinoic acid receptors (RAR, 10,450), and Pregnane X receptor (PXR, 8044). Toxicities associated with NR modulation include hepatotoxicity, cardiotoxicity, endocrine disruption, carcinogenicity, metabolic disorders, and neurotoxicity. These toxicities often arise from the dysregulation of receptors like Peroxisome proliferator-activated receptors (PPARα, PPARγ), the ER, PXR, AR, and GR. This dysregulation leads to various health issues, including liver enlargement, hepatocellular carcinoma, heart-related problems, hormonal imbalances, tumor growth, metabolic syndromes, and brain function impairment. Gene expression analysis using heatmaps for human and rat tissues complemented the functional modulation of NRs associated with the reported toxicities. Interestingly, certain NRs showed ubiquitous expression in tissues not previously linked to toxicities, suggesting the potential utilization of organ-specific NR interactions for therapeutic purposes.

## 1. Introduction

Ensuring the safety of pharmacologically targeted small molecules, particularly during the lead candidate drug discovery phase, involves a comprehensive process integrating findings from in vitro, in vivo, in litero, and in silico studies [1,2,3,4,5]. However, a significant gap persists in understanding the safety implications, especially concerning small molecule interactions with NRs. Common in vitro secondary pharmacology screening assays, such as CEREP, BioPrint^®^ SafetyScreen Panels, and KINOMEscan^®^ (Eurofins Cerep, France), along with custom or proprietary assays, are employed to identify potential off-target interactions. Subsequent investigations employing in vitro functional activity assays may follow the identification of positive or equivocal findings in the initial in vitro screening assays [6,7,8]. Neglecting identified off-target interactions may ultimately lead to adverse findings in nonclinical studies, posing a potentially translatable risk to humans [6,7,8,9,10,11].

In addition to the prediction of off-target interactions and assay hits during the in vitro screening (i.e., Lead Optimization) phase, in vitro absorption, distribution, metabolism, and excretion (ADME) studies are typically conducted, followed by in vivo pharmacokinetics before in vivo pharmacology studies with the parent small molecule [12,13,14]. However, for various strategic reasons, discovery teams may wait until later stages, such as Phase 1 clinical studies, to systematically assess the intended on-target and off-target activity(s) and/or pharmacokinetics of the ‘metabolites’ of the lead candidate drug [15,16,17].

Recent insights from the Innovation and Quality (IQ) Consortium [18] and the New Clinical Development Success Rates Report underscore a notable failure rate for lead candidate drugs in discovery (80%) [1] and clinical studies (94%) [19]. These failures are often attributed to unfavorable interactions with unintended protein targets, including NRs, interactions with the intended target in off-target tissues/cells, unexplainable candidate test article-related advantageous and/or adverse findings likely mediated via non-validated targets for a particular disease, metabolite interactions with unintended targets in intended tissues for efficacious pharmacodynamic responses and/or unintended tissues, or the inadequate physicochemical properties of compounds [20,21,22,23]. Off-target interactions may show weaker affinity than the intended pharmacological target. Their relevance often becomes apparent in the presence of high systemic exposures in toxicology and/or human clinical studies, due to drug–drug interactions, or other unanticipated case examples (e.g., species- and/or sex-specific outcomes) as well as instances of the higher cellular expression of off-target interactions [7,24]. Weaker in vitro affinity (i.e., IC_50_) to off-targets during lead identification and optimization may not necessarily be associated with potential adverse outcomes, considering the strong affinity with the primary intended target (typically in the nM range). However, high cellular expression and drug exposure exceeding the weaker IC_50_ in certain tissues could lead to potentially adverse (sometimes dose-limiting) consequential effects [25].

The recent literature compiled by Alexandar et al. [26] covers the pharmacological context associated with 1900 human targets, including NRs. However, a similar compilation within a toxicology context is currently lacking [27]. A significant gap exists in understanding the safety consequences, particularly concerning small molecule interactions with NRs. The functional modulation of some NRs at toxicological doses has been linked to various toxicities, including Central Nervous System (CNS), hepatic, endocrine, gastrointestinal (GI), and others [28]. The toxicological risks associated with NR interactions are not well described in the literature. NRs are also poorly represented in benchmark secondary pharmacology screening panels. The BioPrint^®^ SafetyScreen panel (Eurofins Cerep, France) has only 4 NRs (AR, GR, Era, and PPARγ) while the Bowes panel [24] and ABBV-70 panels [29] feature only 2 NRs (GR and AR), and the Roche panel [30] has 4 NRs (AR, GR, ER, and PPARγ).

To date, over 300 NRs have been discovered, of which 48 are expressed in humans [31], including orphan receptors. NRs are ligand-activated transcription factors that regulate key functions in reproduction, development, and physiology. The dysregulation of these receptors is often linked to a spectrum of diseases. Because most NRs can be selectively activated or inactivated by small molecules, they are prominent therapeutic targets. Based on sequence analysis, these 48 receptors are classified into seven subgroups: NR0, NR1, NR2, NR3, NR4, NR5, and NR6, with each group containing 2, 19, 12, 9, 3, 2, and 1 receptor(s), respectively [32]. Most approved drugs target two subfamilies, NR1 (PPAR, Vitamin D Receptor (VDR), RAR, FXR, Thyroid Hormone Receptor (THR)) and NR3 (GR, AR, PGR, ER, mineralocorticoid receptor (MCR)). Out of the 48 NRs, only a subset (i.e., 12) has been targeted by small molecule drugs and received Food and Drug Administration (FDA) approval. For example, the GR, AR, Progesterone receptor (PGR), and ER boast 51, 25, 20, and 19 approved drugs (mainly anti-cancer), respectively. Most observed toxicities were not considered significant enough to deter approval due to the risk–benefit criteria.

In this work, we systematically summarized and described the potential nonclinical and clinical safety effects resulting from functional interactions with 44 of the 48 NRs, including knockout (KO) mouse phenotypes. This framework can be used as guiding information for the discovery and development of drugs for non-oncology indications, where NR interactions may result in a significant therapeutic benefit. We utilized curated databases (OFF X™) (Off-X, Clarivate), institutional knowledge, and the available literature to analyze 44 NRs. With the exception of toxicology outcomes for 4 orphan NRs, we analyzed the gene expression of all 48 NRs in humans and selected nonclinical species to identify common expression profiles across different tissues. Furthermore, we organized reported toxicities linked to the functional modulation of the 44 NRs in a tabular and machine-readable format. This compilation was envisaged to serve as a valuable resource for drug discovery and toxicology scientists, providing a foundation to formulate hypotheses to better understand potentially translatable toxicities induced by candidate drugs, whether intentionally designed to target NRs or due to unintentional interactions. Additionally, this information has the potential to contribute to the development of interpretable AI models for the prospective prediction of drug interactions with NRs and their expected toxicities.

## 2. Results

### 2.1. NR Families and Their Toxicological Implications

**Figure 1:**
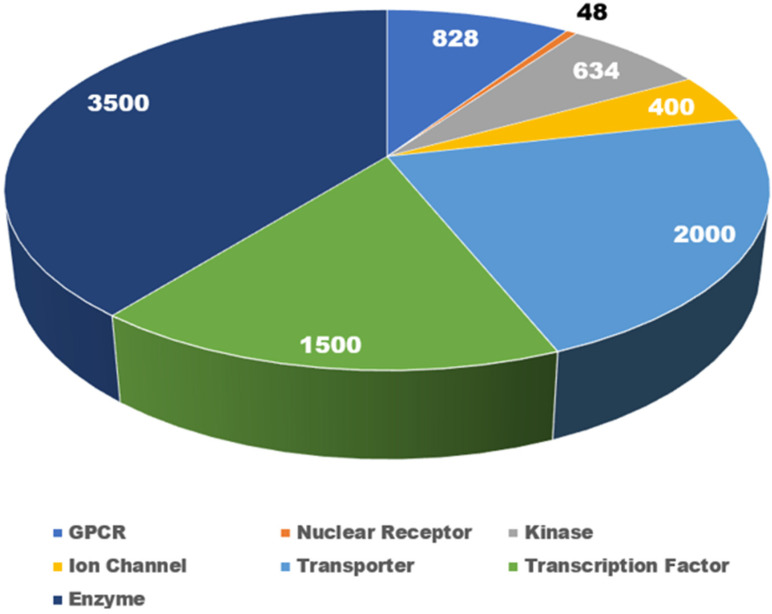
Pie chart showing the approximate number of reported targets in key target families. The number within each pie indicates the number of targets for the respective target class.

Figure 1 presents an overview of major target families, providing a comprehensive count of currently classified drug targets. The human genome, comprising nearly 20,000 protein-coding genes [33], encompasses various target families, including NRs (48) [34], G protein-coupled receptors (GPCRs) (828) [35], kinases (634) [36], ion channels (400) [37], transporters (2000) [38], transcription factors (1500) [39], and enzymes (3500) [40].Despite the NR family having a relatively small number of targets, their functional modulation has been linked to a spectrum of toxicities, including cardiovascular, hepatic, and central nervous system complications.

It is important to note that while certain targets have been the focus of many FDA-approved drugs, not all human-expressed NRs have corresponding approved medications. Among the 48 NRs, only a subset, specifically 13 NRs, received a total of 173 FDA drug approvals (Table 1). Specifically, the GR, AR, PGR, and ER boast 51, 25, 20, and 19 approved drugs, respectively. Furthermore, the isoforms of PPAR (PPARα, PPARδ, and PPARγ) have a total of 21 approved drugs. Additionally, the Small Heterodimer Partner (SHP or NR0B2), THRA, RAR, VDR, Nuclear receptor subfamily 3, group C, member 2 (NR3C2, also known as MCR or Aldosterone receptor), and FXR have 1, 2, 9, 12, 8, and 5 approved drugs, respectively. Table 1 shows the NRs along with the number of approved drugs for each NR. However, the remaining NRs currently lack approved drugs. Nevertheless, the literature lacks an integrated context of the toxicological aspects associated with the exaggerated pharmacology of both NRs with approved drugs and those without. To address this gap, we have compiled information on toxicities associated with NRs using manually curated databases such as Off-X (Clarivate).

### 2.2. Small Molecules and Nuclear Receptor Interactions: Safety Alert Analysis

We conducted an in-depth analysis focused on small molecules, including those both approved and in discovery/development stages, and their interactions with NRs across 92,858 safety alerts. Among these alerts, the top five targets associated with the highest number of alerts are the GR (18,328), AR (18,219), ER (12,028), RAR (10,450), and PXR (8044). Further details can be found in Supplemental Excel Table S1, which provides comprehensive information on all 92,858 safety alerts. This includes their associated targets, drugs, references, and adverse events.

### 2.3. Detailed Analysis of Select 44 NRs

**Figure 2:**
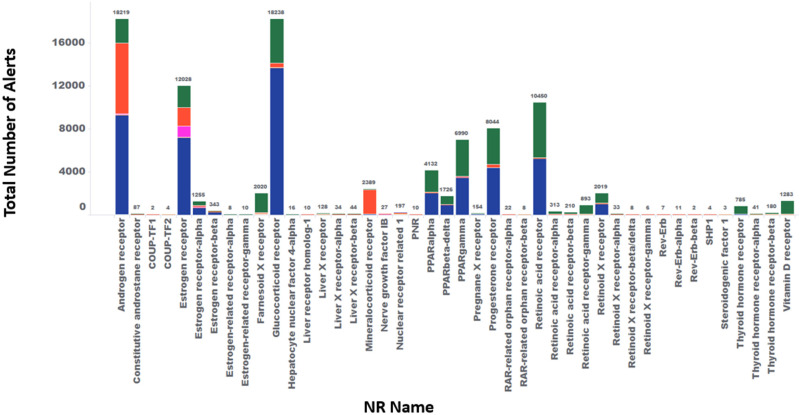
Number of alerts associated with each of the 44 NRs. Green indicates agonists, red indicates antagonists, magenta indicates degraders, and blue indicates modulators (either agonist, antagonist, or partial agonist). The X-axis represents the name of the NR, and the Y-axis shows the number of alerts.

Figure 2 shows the overall count of safety alerts connected to 44 different NRs, with the highest number of safety alerts associated with the AR, GR, ER, RAR, and PXR. This presents the number of drug molecules being developed, the sources of published alerts, the number of alerts in various stages of discovery and development, references, and the top adverse outcomes. Primarily due to the limited publicly available information on discovery efforts focused on the four orphan NRs, we were unable to capture related adverse events.

#### 2.3.1. AR

Among the 92,858 alerts, 18,219 are relevant to 69 drug molecules acting on the AR. Of these, 9182 alerts are from regulatory agency communication, indicating that some AR-acting drugs received approval despite significant associated toxicities, likely due to their efficacy in addressing unmet oncology needs. These alerts span various developmental stages, with notable phases including post-marketing (4191), Phase III (2752), Phase II (2268), Preclinical (1393), Phase IV (127), and Target Discovery (69). Key adverse events reported include fatigue, hypertension, nausea, diarrhea, hot flush, rash, decreased appetite, carcinogenicity, testicular atrophy, asthenia, headache, and weight loss. Additionally, analysis of KO mice data revealed that hemizygous mutant males lacked AR-exhibited androgen resistance, leading to small undescended testes and the absence of epididymal structures, vas deferens, and male accessory glands. Despite displaying physical and behavioral traits resembling females, they lack female reproductive organs, and the findings were consistent with the testicular toxicity observed in clinical/post-marketing trials (Supplemental Excel Table S1).

#### 2.3.2. GR

Out of the 92,858 alerts, 18,238 involve 88 drug molecules targeting the GR, with 7902 alerts documented in regulatory agency communication reports. These alerts span multiple developmental stages, including post-marketing (4617), Phase III (1570), Phase II (982), Preclinical (1950), Phase IV (152), and Target Discovery (101). Notable adverse events include glaucoma, headache, hypertension, hyperglycemia, abnormal glucose metabolism, weight gain, nausea, adrenal insufficiency, vomiting, and depression. Furthermore, the KO mice database showed that homozygous null mutants are characterized by underdeveloped lungs, enlarged adrenals, elevated serum corticosterone and ACTH levels, and impaired adrenaline synthesis. Mice carrying a point mutation showed impaired gluconeogenesis and erythropoiesis. These effects are consistent with clinically observed adrenal and abnormal glucose effects noted in clinic due to GR-acting drugs. Furthermore, the KO mice database showed that homozygous null mutants are characterized by underdeveloped lungs, enlarged adrenal glands, elevated serum corticosterone and ACTH levels, and impaired adrenaline synthesis. Mice carrying a point mutation exhibited both impaired gluconeogenesis and erythropoiesis. These effects are consistent with clinically observed adrenal abnormalities and glucose dysregulation noted in clinics due to drugs acting on the GR.

#### 2.3.3. ER (α and β)

Of the 92,858 alerts, 13,644 are associated with 74 drug molecules targeting ER alpha (ERa) and ER beta (ERb), with 4817 alerts documented in regulatory approval packages. These alerts span various developmental stages, including post-marketing (2156), Phase III (1536), Phase II (1971), Preclinical (1470), Phase IV (6), and Target Discovery (218). Key adverse events include carcinogenicity, nausea, fatigue, vomiting, headache, diarrhea, breast cancer, arthralgia, hot flush, anemia, and constipation.

#### 2.3.4. RAR (α, β, and γ)

Out of the 92,858 alerts, 11,866 involve 18 drugs acting on RARs, including alpha, beta, and gamma subtypes, with 4483 alerts documented in regulatory approval packages. These alerts span various developmental stages, including post-marketing (3107), Phase III (467), Phase II (488), Preclinical (1053), Phase IV (31), and Target Discovery (159). Key adverse events include headache, erythema, dry skin, pruritus, depression, cheilitis, teratogenicity, arthralgia, myalgia, alopecia, and nausea.

#### 2.3.5. PXR

Of the 92,858 alerts, only 154 involve 102 drugs targeting the pregnane X receptor (PXR), with 152 alerts sourced from journal articles and 2 from congress alerts, and none documented in regulatory approval packages at the time of this publication. These alerts span various developmental stages, including post-marketing (42), clinical/post-marketing (14), Phase I (3), Preclinical (73), and Target Discovery (22). Notable adverse events include endocrine disorders, increased cholesterol, increased LDL, hepatotoxicity, and hepatomegaly.

#### 2.3.6. PPAR (α, γ, and δ)

Among the 92,858 alerts, 12,848 involve 66 drugs targeting PPARs. These alerts span various developmental stages, including post-marketing (3022), Phase III (1171), Phase II (1223), Preclinical (1795), Phase IV (34), and Target Discovery (229), among others. Key adverse events associated with PPAR modulation include carcinogenicity, headache, nausea, diarrhea, edema, weight gain, abdominal pain, dizziness, hepatotoxicity, and drug-induced liver injury (DILI).

#### 2.3.7. NR3C2 (Also Known as the Mineralocorticoid Receptor)

Out of the total alerts, 2389 are linked to 20 drugs targeting the NR3CR2 (also known as the mineralocorticoid receptor). These alerts span various developmental stages, including post-marketing (380), Phase III (262), Phase II (240), Phase I (92), Preclinical (368), Phase IV (6), Target Discovery (32), and others. Notable adverse events include hyperkalemia, headache, gynecomastia, hypotension, nausea, dizziness, carcinogenicity, vomiting, and diarrhea.

#### 2.3.8. LXR (α and β)

Of the total alerts, only 206 involve 11 drugs targeting liver X receptors (LXR). These alerts span various developmental stages, including clinical/post-marketing (10), Phase I (25), Preclinical (43), Target Discovery (128), and others. Key adverse events include abdominal oocyte maturation, female sterility, increased cholesterol, intestinal toxicity, ovarian hyperstimulation syndrome, reduced visual function, squamous cell carcinoma of the lung, hypertriglyceridemia, and age-related macular degeneration.

#### 2.3.9. NR5A1 (Steroidogenic Factor 1)

NR5A1 has only been associated with three alerts, all related to genetic variants linked to disorders of sex development, male infertility, and primary ovarian failure.

#### 2.3.10. NR1D1 (Rev-Erb)

NR5A1 has been annotated with 20 safety alerts, sourced from the small molecule antagonist SR-8278 and genetic knockdown or KO. Key toxicities include nervous system disorders, colitis, fulminant hepatitis, muscle atrophy, fibrosis, and noninfective encephalomyelitis, all sourced from peer-reviewed publications.

#### 2.3.11. NR4A1 (Nerve Growth Factor IB)

NR4A1 has been annotated with 27 safety alerts, including gene expression inhibitors and variations in expression levels. Key toxicities include autoimmune disorders, decreased regulatory T cells, angiopathy, hypertension, bipolar disorder, gingival hypertrophy, schizophrenia, and renal hypertension, sourced from journal publications.

#### 2.3.12. VDR (Vitamin D Receptor)

Of the total alerts, 1283 involve 14 drugs targeting the vitamin D receptor (VDR). These alerts span various developmental stages, including post-marketing (134), Phase III (30), Phase II (74), Phase I (64), Preclinical (200), Phase IV (6), Target Discovery (58), and others. Key adverse events include hypercalcemia, carcinogenicity, nausea, contact dermatitis, pruritus, affected normal visual function, hyperphosphatemia, rash, vomiting, and diarrhea.

#### 2.3.13. THRA and THRB (Thyroid Hormone Receptors)

Among the 92,858 alerts, 1006 involve 23 drugs targeting thyroid hormone receptors (THRA and THRB). These alerts span various developmental stages, including post-marketing (240), Phase III (134), Phase II (103), Phase I (63), Preclinical (149), Target Discovery (46), and others. Key adverse events include endocrine disorders, diarrhea, nausea, headache, insomnia, fatigue, drug interactions, weight loss, abdominal pain, and arthralgia.

#### 2.3.14. NR1I3 (Constitutive Androstane Receptor)

Out of the reviewed alerts, 87 pertain to 13 drugs in conjunction with NR1I3 KO mice. Noteworthy adverse events identified include carcinogenicity, dyslipidemia, glucose intolerance, hepatic steatosis, obesity, endocrine disorders, liver toxicity, liver tumors, biliary adverse events, and cystic bile duct hyperplasia.

#### 2.3.15. NR5A2 (Liver Receptor Homolog 1)

NR5A2 has been associated with only 10 safety alerts. The key toxicities include glucose intolerance impairment, hepatic steatosis, liver injury, increased liver weight, weight gain, and hyperphagia (i.e., sourced from journal publications).

#### 2.3.16. NR2F1 and NR2F2 (COUP-TF1 and COUP-TF2)

Both NR2F1 and NR2F2 have been annotated with only six safety alerts, including visual impairment, renal cysts, and a bicuspid aortic valve.

#### 2.3.17. NR0B2 (SHP1)

NR0B2 has been associated with only 4 alerts that pertained to protection against viral hepatitis-related liver cancers and the repression of bile acid biosynthesis (i.e., via downregulation of Cyp7a1) [41].

#### 2.3.18. RXR (α, β, and γ)

Among the 92,858 alerts, 2066 are associated with 8 agonists and several conditional knockouts of RXR isoforms, with 834 alerts documented in regulatory communications. These alerts span various developmental stages, including post-marketing (326), Phase III (34), Phase II (182), Phase I (64), Preclinical (266), Target Discovery (78), and other phases. Key adverse events linked to RXR modulation include headache, hypothyroidism, teratogenicity, hypertriglyceridemia, hyperlipidemia, neutropenia, rash, nausea, and leukopenia.

#### 2.3.19. Human, Rat, Mouse, Dog, and Monkey mRNA Expression of NR

**Figure 3:**
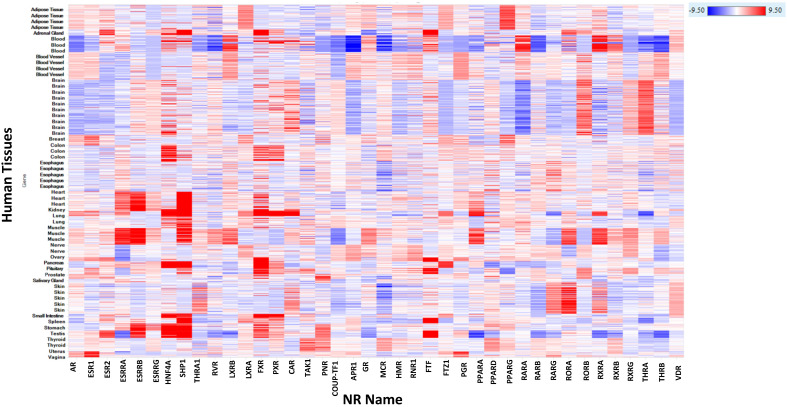
Heatmap of human mRNA expression of NRs. The color gradient from red to blue indicates high to low expression levels. The X-axis represents the NR name, and the Y-axis shows the tissues where the expression is noted. The heatmap is colored using log_2_(FPKM + 0.1) values by tissues, ranging from −9.5 (red) to 9.5 (blue).

In Figure 3, we present a heatmap illustrating human mRNA expression of the 48 NRs described in Supplemental Excel Table S1. This heatmap effectively highlights key tissues where toxicities are implicated due to the functional modulation of these receptors. Interestingly, some receptors show ubiquitous expression in other tissues where no toxicities have been reported, suggesting these tissues may be of therapeutic interest.

**Figure 4:**
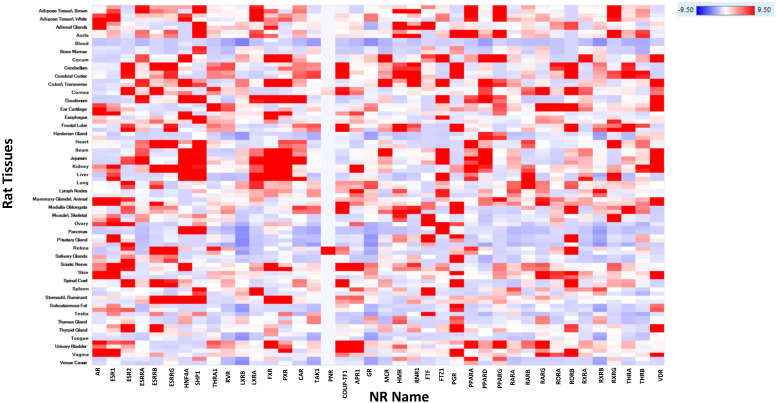
Heatmap of Rat mRNA expression of NRs. The color gradient from red to blue indicates high to low expression levels. The X-axis represents the NR name, and the Y-axis shows the tissues where the expression is noted. The heatmap is colored using log_2_(FPKM + 0.1) values by tissues, ranging from −9.5 (red) to 9.5 (blue).

Figure 4 shows the heatmap of rat mRNA expression for the 48 NR targets. There is notable concordance in mRNA expression profiles between rats and humans, reinforcing the translational potential of the findings and emphasizing the utility of rat models in predicting potential human toxicities related to NR modulation.

Additionally, Supplemental Figures S1–S3 present the expression of these receptors in mice, dogs, and cynomolgus monkeys.

### 2.4. Statistical Analysis of Relative Fold Change Expression in Various Tissues for NR

The median expression level of each of the available 38 NRs across 52 human tissues is detailed in column 2 of Supplemental Table S2. NR expression across these tissues varies significantly, ranging from as low as 0.015 to as high as 89.5 (in FPKM).

**Figure 5:**
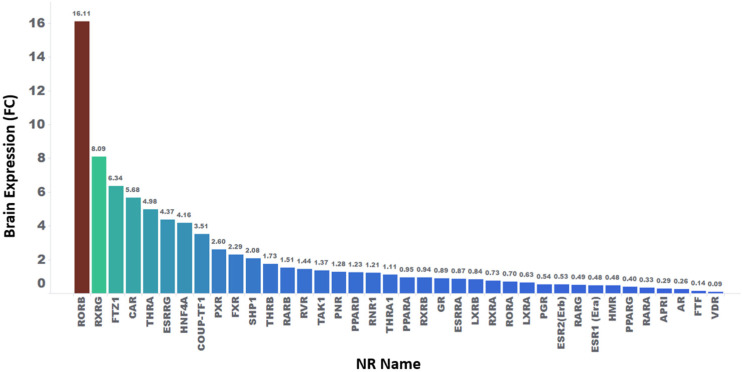
Computed mean brain fold expression of each NR relative to the median expression across all tissues. A higher number indicates relatively higher mean expression in the brain. Red to blue represents high to low mean expression in the brain.

Supplemental Table S2 also includes the relative fold change (FC) expression of these NRs in various tissues. This FC calculation involves dividing the mean expression of a given NR in each tissue by the overall median expression of a given NR across tissues (i.e., mean expression in a given tissue/median expression across tissues). A fold change greater than 1.2 is considered significant, indicating higher relative expression in a specific tissue. For example, Figure 5 illustrates the relative fold expression in the brain for NRs with available expression data. Similar relative fold change values for an additional 29 human tissues, including key tissues such as the liver, cardiovascular system, kidneys, testis, lungs, and stomach, are provided in Supplemental Table S2.

According to our established FC criterion, 17 NRs show mRNA expression levels higher than 1.2 times the median expression (Supplemental Table S2). Nonetheless, if the fold change is less than 1, indicating that the mRNA expression may be lower than the median, in some instances, the lower fold expression may still be consequential. Additionally, mRNA–protein correspondence is not readily available to properly infer correlations between mRNA and protein expression levels for many of the NRs.

## 3. Discussion

The assessment of drug safety requires good laboratory practice (GLP) testing in both in vitro systems as well as preclinical toxicology species before advancing to first-in-human trials [5,42,43,44]. The detection of toxicity varies by species, sex, organ, and cell-type but the absence of toxicity in two species often strongly correlates with humans [45,46]. Numerous reports indicate that human adverse drug reactions (ADRs) and related attrition predominantly involve cardiovascular (CV) [47], hepatic [48], and neurological toxicities [49] in that order [50]. Moreover, Weaver et al. [51] demonstrated the significant contribution of the CV, liver, and CNS to attrition and ADRs throughout drug discovery and development. While other toxicological categories, such as immunological, photosensitivity, dermatological, gastrointestinal, musculoskeletal, respiratory, oncological, hematological, bone marrow, renal, reproductive, and genetic, also contribute to overall adverse events (AEs), their contributions are generally less pronounced; however, the precise contribution of off-target interactions (or which target family is most commonly linked to drug attrition) remains unclear. Nonetheless, evidence suggests that highly promiscuous (demonstrating off-target interactions)-type drugs are more likely to be discontinued during clinical development or withdrawn from the market [22,52]. To address this drug attrition concern, several in vitro off-target screening panels have been described in the literature with the goal of developing less promiscuous compounds and deprioritizing compounds that show activities on identified off-targets [24,29,30,53,54,55,56,57]. These panels encompass targets from various target families, including representatives from NRs, with defined organ toxicities associated with the modulation of each in the panel. In the following section, we outline various safety screening panels and the diverse target families included in each panel for early derisking.

### 3.1. Improving Drug Safety through Early Target Panel Screening

**Figure 6:**
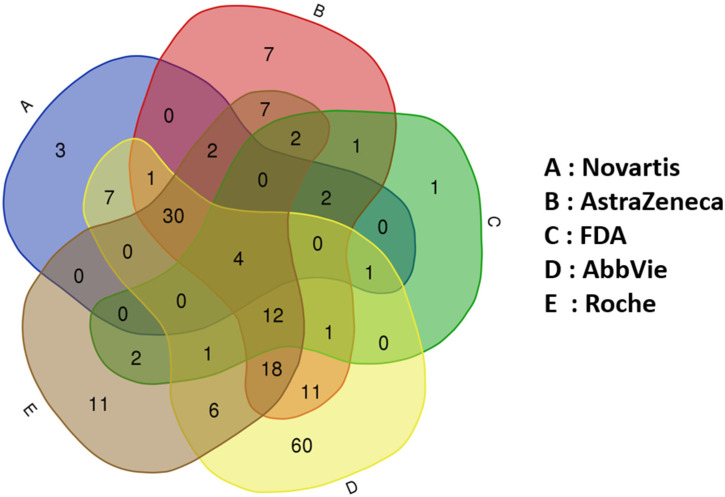
Venn diagram of published safety screening panels by pharmaceutical companies and the FDA. The names of each panel are shown as A, B, C, D, and E. Corresponding companies or the FDA are labeled separately.

Over the past decade, pharmaceutical companies and the FDA have advocated for safety-relevant targets for early safety screening to enhance the success probability of lead compounds in both nonclinical and clinical studies. Figure 6 illustrates a Venn diagram of these panels, highlighting common targets, with some targets represented three times, implying different functions (antagonist, agonist, or inverse agonist). It is crucial to acknowledge that the functional modulation of each target, whether it acts as an antagonist, agonist, inhibitor, partial agonist, or modulator, leads to diverse toxicological consequences.

Supplemental Table S2 presents a comprehensive comparison of target panels by four pharma companies and the FDA. The column labeled “Target-action” delineates the functional activity of the targets suggested by each company or the FDA. Columns 2–6 display the names of companies or the FDA, and the last column reflects the consensus from these panels. A “1” in cells indicates that the company has considerations or suggestions, while 0 in the cells indicates no suggestion from a particular company or the FDA. For instance, three targets show unanimous agreement across all panels, indicating four functional modulations each (5HT2B agonism, GABAA-antagonism and -agonism, and SERT inhibition), resulting in a target consensus score of 5. Another critical example is the AR, a NR, with both antagonism and agonism suggested by three pharmaceutical companies (AstraZeneca, AbbVie, and Roche) and the FDA, resulting in a target consensus score of 4. Other NRs considered include the GR (score of 3), PPARγ (score of 3), and the ER (score of 1). In total, only four NRs (AR, GR, PPARγ, and ER) are part of one or more suggested panels, indicating a lower presence compared to other target families like GPCRs, despite the larger toxicological role associated with NR modulation. Nevertheless, in this work, we have provided safety alerts for 44 of 48 NRs, including the 4 NRs that are part of these published panels. These additional supplemented NRs and associated outcomes can be used to contextualize in vivo findings or derisk early compounds interacting unintentionally with NRs.

### 3.2. NRs: Roles, Safety Risks, and Therapeutic Potential

NRs, which function as ligand-dependent transcriptional regulators, are abundant in animals and share both structural and functional similarities [34,58,59]. In the absence of ligands, these receptors primarily exist as monomers and form complexes with chaperones to maintain cytoplasmic homeostasis [60,61,62]. Functionally, NRs engage in critical physiological programs, including embryonic development, the maintenance of differentiated cellular phenotypes, metabolism, and cell death. Dysfunctions in NR signaling lead to proliferative, reproductive, and metabolic diseases such as cancer, infertility, obesity, and diabetes [63,64]. The druggable nature of NRs has made them attractive targets for various human diseases, resulting in the development of receptor-selective full, partial, and inverse agonists, as well as compounds that activate only a subset of functions induced by the cognate ligand, and molecules that act in a cell-type-selective manner [65,66]. However, many of these drugs have various adverse effects due in part to promiscuity in NR targeting, signal regulation, or transcription of downstream gene targets.

The classification of NRs is based on the types of ligands they bind to, and they are divided into seven main groups: NR0, NR1, NR2, NR3, NR4, NR5, and NR6 [34]. The initial category includes steroid hormone receptors like GRs and ERs, which regulate downstream transcription processes through homodimer structures [67]. The second category encompasses nonsteroidal hormone receptors such as PPARs, TRs, RARs, the AR, FXR, and others. These receptors typically form heterodimers with RARs to control the transcription and expression of downstream genes. The final two categories consist of orphan receptors lacking known endogenous ligands. Most of these NRs can bind to their respective DNA-responsive elements (DREs) either as monomers or homodimers to regulate transcriptional expression. Below, we discuss specific NRs and their toxicological outcomes [68,69,70]. Given the impracticality of discussing toxicities across all tissues due the to modulation by these NRs discussed within this section (although all are listed in Supplemental Table S1), we choose to concentrate our discussion on adverse data concerning key organ toxicities, such as those affecting hepatic, CNS, and CV tissues. Additionally, we have separately included extensive discussions on PPARα and γ, the GR, and AR, as these are associated with adverse alerts and are also part of published safety panels.

### 3.3. Integrative Insights into Small Molecule Interactions with NRs

**Figure 7:**
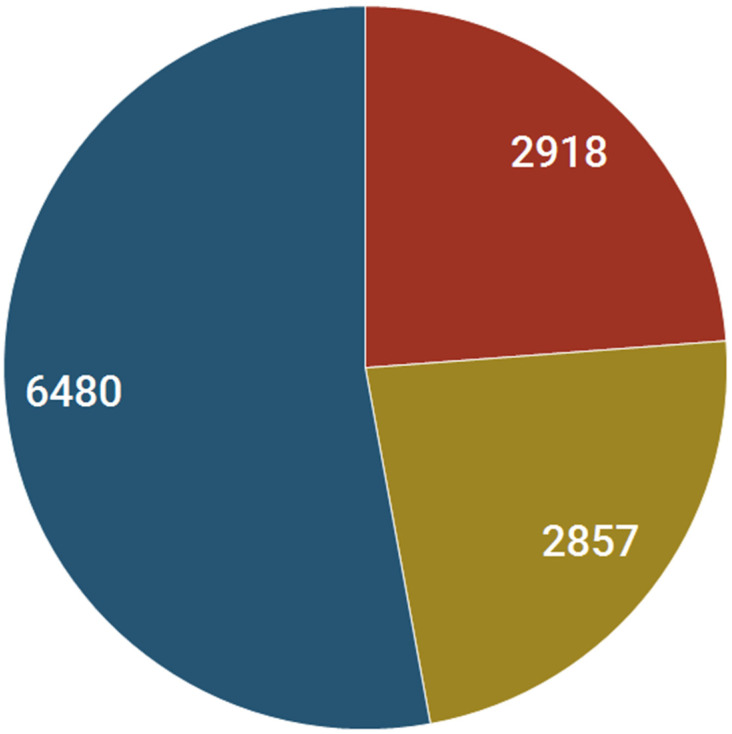
Pie chart showing the number of annotated CNS alerts (blue), CV alerts (red), and Hepatic alerts (beige).

Although our analysis indicates that NR interactions are involved in inducing a spectrum of toxicities, we noted a total of 12,255 alerts linked to hepatic, CNS, and CV toxicities involving 39 NRs. Of these, 6480, 2918, and 2857 are related to CNS, CV, and hepatic issues, respectively (see Figure 7). The top NRs with the highest CNS alerts are the AR (1733), GR (1035), PPARγ (346), PXR (642), and RAR (528). A similar trend is observed for hepatic and CV toxicities as well. Nevertheless, the top toxicities associated with NRs are not limited to these three organs. Instead, the GI (8515) tract and the skin (8162) are linked to a higher number of alerts than these two organ’s toxicities. Furthermore, reproductive toxicities (4955), mucosal connective disorders (4394), and vascular disorders (3137) are more prevalent than hepatic and CV toxicities, implying that NRs generally do not fall into key canonical organ toxicity categories (i.e., hepatic, CV, and CNS).

It should be noted that drugs designed for non-NR targets or those intended to selectively interact with a specific NR can also engage different NRs as off-targets with varying potencies. For instance, Rao et al. [29] devised a drug repurposing methodology to computationally screen small molecule interactions including 41 of the 48 NRs using AI/ML-based off-target interaction prediction for small molecules. These predictions identified a total of 1293 interactions, involving 511 drug molecules from the pool of 2766 FDA-approved drugs under investigation. Moreover, the method where 10 or more out of the 2766 FDA-approved drugs with confirmed in vitro assay data indicated interaction(s) with 13 NRs, including the AR, GR, Progesterone receptor (PGR), Estrogen receptor 1 (ESR1), NR subfamily 1 group I member 2 (NR1I2), Estrogen receptor beta 2 (ESR2), NR3C2), Thyroid hormone receptor alpha (THRA), Vitamin D3 receptor (VDR), PPARα, PPARγ, Thyroid hormone receptor beta (THRB), and Bile acid receptor (NR1H4). Intriguingly, 98, 95, and 92 drugs interacted with the AR, PGR, and GR, respectively. This suggests that these NR targets are modulated by numerous FDA-approved drugs with varying potencies, even though many are not intended to interact with these NRs. Additionally, the functional modulation of these 13 targets has been associated with several toxicological outcomes, as illustrated in Supplemental Excel Table S1, particularly for those NRs with a high number of adverse alerts.

### 3.4. Comprehensive Analysis of Hepatic, CNS, and CV Toxicities

#### 3.4.1. Hepatic Toxicity

More than 100 adverse terms have been curated to describe 2918 hepatic toxicity alerts (Off-X, Clarivate). Additionally, some of the annotated adverse terms represent the same thing, such as ‘hepatotoxicity’ and ‘liver toxicity’. Nevertheless, after removing redundant terms, we observed that the top annotated liver toxicities are DILI (386), Jaundice (121), Hepatitis (110), Cholestasis (65), Hepatomegaly (56), and Hepatic steatosis (50). Furthermore, 10 alerts are linked to idiosyncratic DILI, which differs from intrinsic DILI. Overall, the 2918 liver toxicity alerts involve one or many of the 31 NRs (of 44 NRs analyzed in the work). The top NRs linked to the liver alerts are the AR, PPARγ, PPARα, ER, GR, PXR, FXR, and RXR. It is interesting to note that the AR, GR, ER, and PPARγ are already part of various early derisking panels (Table 2), including our own. Rao et al. (2023) introduced an AI/ML-based predictive model for DILI, where they identified 43 off-target interactions (with one or many of these off-targets) linked to potential DILI in small molecules. Among these off-targets were four NRs: the AR, PGR, PPARγ, and ESR1, suggesting the role of NRs is in agreement with the higher number of hepatotoxicity alerts associated with these specific NRs.

#### 3.4.2. CNS Toxicity

Our analysis identified 2918 CNS alerts involving one or more of 32 NRs. The top NRs, whose functional modulation led to these CNS alerts, are the AR (1733), GR (1035), ESR (791), PGR (643), and RAR (528). However, the latter three are not included in established safety in vitro screening panels, perhaps due in part to the low level of expression relative to other NRs in the human brain (Figure 5). Interestingly, none of the NRs whose expression is highly enriched in the brain were among the NRs with the most alerts. Considering the proposed roles of NRs like the RXR and ROR in CNS regeneration and neuroinflammation, a favorable safety profile would increase the attractiveness of those NRs as therapeutic targets for diseases like multiple sclerosis and Alzheimer’s disease [71].

The CNS alerts originate from over 200 defined adverse terms documented in the literature. The most prominent CNS toxicity alerts include headache (1457), dizziness (767), stroke (162), seizure (158), convulsions (58), dysgeusia (116), syncope (108), somnolence (86), and peripheral neuropathy (65). Most of these CNS toxicities manifest during clinical trials, suggesting that many may be missed during preclinical studies. However, seizures, convulsions, somnolence, and peripheral neuropathy stand out as toxicities that are generally monitored in nonclinical settings. For example, we may seek to understand how frequently headaches correlate with seizures. However, since headaches are not typically monitored as a top CNS alert, establishing such connections becomes difficult; this is consistent with our analysis, where we were only able to capture a limited number of preclinical CNS alerts on convulsions/seizure.

#### 3.4.3. CV Toxicity

Several adverse events related to cardiac disorders have been identified and documented in various sources from the literature. Examples of well-described terms include cardiac arrhythmias, cardiac neoplasms, cardiac valve disorders, coronary artery disorders, endocardial disorders, heart failure, myocardial disorders, and pericardial disorders. Furthermore, each of these terms encompasses several subcategories of adverse events. Therefore, attempting to associate NR modulation with all these terms is not practical. Nevertheless, our analysis suggests that 2918 CV toxicity alerts involve one or more of the 22 NRs. These alerts encompass 122 distinct cardiac disorder terms. The most prominent CV toxicity alerts include cardiac failure (293), myocardial infarction (241), palpitations (137), arrhythmia (128), congestive cardiac failure (127), atrial fibrillation (118), coronary artery disease (102), and tachycardia (88). The primary NRs associated with these CV toxicity alerts are the AR (790), PPARγ (648), GR (468), ESR (316), and PPARα (124). Although these top NRs are included in some established in vitro safety screening panels as detailed above, we have also identified the roles of an additional 17 NRs linked to CV toxicity (see Supplemental Excel Table S1). The activity of these additional NRs, when assessed, can provide valuable context for observed in vivo CV toxicity.

#### 3.4.4. PPARa and g, AR, and GR

The biological activities associated with PPARs are intricately linked to the adverse effects of drugs, particularly those involving PPAR ligands or xenobiotic chemicals. Such associations often lead to toxicity across various tissues, necessitating a comprehensive understanding to navigate the complex web of interactions and grasp the impact of PPAR functional modulation [72,73,74,75].

In cardiac muscles, for example, where PPARs show high expression and play a pivotal role in metabolic disorders and endocrine disruption, interference with these receptors can disrupt metabolic homeostasis and hinder the development of the cardiovascular system [76,77,78]. Similarly, in the liver, PPARs are vital for fatty-acid and glucose metabolism [79,80]. Disruption by xenobiotic chemicals can induce metabolic disorders, primarily through the activation of PPAR subtypes, notably PPARα. This subtype is recognized as a target for pollutants that interact and induce metabolic disorders, thereby disrupting liver homeostasis.

Rao et al. [81] contributed to this understanding by developing AI/ML predictive models, identifying 43 key targets as discriminating factors for compounds with the most and no DILI, including two NRs, PPARγ and the AR. Pittenelli and Videla [82] explored the reported measured liver PPARγ mRNA levels in obese patients with steatosis and steatohepatitis, suggesting a mechanism linking liver steatosis and elevated PPARγ levels, this further increasing liver toxicities such as DILI, consistent with the findings of Rao et al. [27].

In the gastrointestinal (GI) tract, especially the intestine and colon, which express high levels of PPARγ, this receptor is closely linked to GI injury and inflammation [83,84]. Disruption of GI homeostasis by internal or external factors leads to the release of pro-inflammatory cytokines, mediated by PPARs and their ligands. Specifically in the colon, PPARγ downregulates NF-κB and MAPK signaling pathways, inhibiting the production of inflammatory cytokines, offering a potential therapeutic target for GI diseases.

In the reproductive system, abnormal regulation triggered by exposure to compounds, whether endogenous or exogenous, can lead to physiological dysfunction [85,86,87]. Specific drugs acting as PPAR agonists, such as Thiazolidinediones (TZDs), a class of PPARγ agonists, have been associated with fluid retention and heart failure [88,89]. Similarly, glitazones, another PPARγ ligand, have been reported to cause peripheral edema, congestive heart failure, and weight gain [90,91,92]. Gemfibrozil, a PPARα agonist, has shown associations with tumorigenesis, muscle weakness, and the liver [93,94,95]. Troglitazone, a PPARγ agonist, was withdrawn due to potential DILI [96,97].

Clinical studies suggest that troglitazone’s hepatotoxicity results from a combination of metabolic and nonmetabolic factors, involving complex drug–protein interactions in the liver and biliary system, in agreement with DILI AI/ML produced by Rao et al. [81]. In the clinical context, rosiglitazone, a PPARγ agonist used for diabetes, has been associated with various adverse effects, including lactic acidosis, cardiac failure, and weight gain, highlighting the need for transparency in adverse event reporting to ensure a thorough understanding and assessment of drug safety profiles.

Similarly, significant documentation disparities exist for drugs targeting PPARα. For instance, pemafibrate, an approved drug, is associated with confirmed adverse effects, while discontinued drugs like MK-0767 have none reported. This emphasizes the importance of comprehensive adverse event reporting.

For drugs targeting the AR, there is relatively high expression in various human tissues, including the liver, blood vessels, prostate, vagina, heart, testis, and adipose tissue. This indicates significant interactions in these tissues, impacting both toxicology and efficacy. The functional modulation of the AR is linked with confirmed alerts including fatigue, hypertension, nausea, and others. Clinical studies further support these findings, consistent with AI/ML-based predictive models for DILI reported by Rao et al. [81].

Several drugs targeting the GR have been associated with significant toxicities, whether on-target effects or off-target interactions. For example, the glucocorticoid Ciclesonide has been associated with over 100 alerts, emphasizing the importance of screening compounds which may inadvertently reveal GR modulating in the early drug discovery stages to minimize late-stage candidate drug attrition. Toxicities associated with drugs acting on GR include glaucoma, headache, hypertension, and others, underscoring the need for a thorough understanding and assessment of drug safety profiles (Supplemental Excel Table S1).

The regulation of gene expression by NR is crucial for metabolism, cancer, neurological diseases, development, and immune responses, with key target genes, such as those for the GR, AR, PGR, ER, and PPARγ, governing specific pharmacological effects in particular tissues. Examples of GR targets include FKBP5, TSC22D3, SGK1, PCK1, and PER1, while AR targets encompass KLK3, TMPRSS2, FKBP5, NKX3-1, and SARDH. PGR targets involve IGFBP1, MUC1, HAND2, FOXO1, and SERPINB2, and ER targets include GREB1, TFF1, MYC, and BCL2. PPARγ targets consist of FABP4, ADIPOQ, CPT1A, SCD, PPARGC1A, PGK1, and PRKCZ. For example, Venkatachalam et al. [98] computationally identified 1154 PPARγ direct target genes and constructed a PPARγ disease gene network, revealing 138 target genes associated with 65 unique diseases. The network indicates strong associations with cancer and neurological diseases, with 38 direct target genes involved in prostate cancer. The study validated two key PPARγ target genes, PRKCZ and PGK1, which were repressed upon PPARγ activation by its natural ligand, 15d-PGJ2, in three prostate cancer cell lines. A clinical case report described a child presenting hematologic and central nervous system anomalies due to a mutation in PGK1 [99], implying abnormalities associated with the disruption of the PPARγ network. Additionally, a next-generation sequencing study in 20 patients with cerebral palsy found that 11 patients (55%) had pathogenic or potentially pathogenic variants, including one patient with a mutation in the PGK1 gene, indicating that a disrupted PPARγ network can lead to abnormalities. Another study found that the deletion of the PRKCZ gene in a genome-wide association study increased the risk of thromboembolic stroke in patients with atrial fibrillation [100]. Overall, understanding their gene regulatory mechanisms is critical for clearly contextualizing pharmacology and toxicology associated with NRs in select tissues. However, in our literature analysis, we have not considered the mechanistic role of these specific NR-targeted gene interactions.

#### 3.4.5. Limitations in NR Modulation Analysis

Although analyses utilizing data mining approaches such as Off-X (Clarivate) and literature reviews help in summarizing complex organ toxicities associated with NR functional modulation, these analyses are inherently biased by the existing knowledge base and often overlook potential institutional knowledge. Furthermore, they may omit crucial toxicological information from early discontinued drugs by pharmaceutical companies, given that these companies generally do not publish preclinical toxicology results. Additionally, the process of annotating adverse alerts is largely manual, and not all NRs and toxicological relationships may be fully covered within context. Furthermore, another limitation of this study is the potential for bias in the alerts data, as not all NRs have received extensive investigation, unlike well-known ones such as the AR, GR, ER, PGR, and PPARs. Consequently, we noted a higher number of alerts associated with these extensively studied NRs. Nevertheless, we anticipate a greater number of alerts for lesser-studied NRs as well as we develop new drugs targeting these.

When interpreting Supplemental Excel Table S1 and other discussed toxicities in this work, it is essential to understand that not all adverse alerts carry the same weight, and each one needs to be understood within the framework of therapeutic indication and biological relevance. Particularly in oncology indications, some alerts may not be concerning, given the potential benefit-to-risk ratio, but the same alerts may be significant for immunology. Additionally, for the safety assessment of any new compounds, relying solely on in vitro activities with NRs is inadequate for indicating adverse alerts. Factors such as systemic exposure levels, brain exposure (if the toxicity is CNS-based), the volume of distribution, drug tissue distribution, tissue expression of the relevant NRs across species, and the residence time of a drug with particular NRs must be considered before fully utilizing Supplemental Excel Table S1.

It is important to highlight that the data presented are from public sources. The raw clinical data sets are not available publicly, deterring the opportunity for meta-analysis, such as with the revised version of the Cochrane tool, known as RoB 2. Thus, the data mining evaluation presented here, and similar analyses in the literature, are susceptible to biases and may contain inaccuracies. Therefore, it is crucial to consult multiple independent data analysis reports, along with input from internal subject matter experts if available, when assessing potential adverse alerts due to NR modulation.

Furthermore, understanding concepts like risk of bias and publication bias is essential for critically evaluating and synthesizing evidence from this analysis in linking adverse events with certain NR modulations. Risk of bias refers to systematic errors that can affect study validity, while publication bias occurs when studies with positive results are more likely to be published (while negative studies may not be published), leading to significant bias. Incorporating these concepts certainly helps ensure robust, reliable, and applicable findings, ultimately leading to better-informed NR derisking decisions. However, since our work relied on a large number of published adverse event reports (including regulatory agency communications) linked to NR modulation, it is impractical to assess the validity of all publicly available information. Additionally, in Supplemental Table S1, we have provided references for all adverse outcomes linked to the studied NRs. Therefore, when utilizing this work for early derisking, we suggest employing a weight-of-evidence (WoE) approach, including institutional knowledge, and not solely relying on the results presented here. Additionally, the PICO (Population, Intervention, Comparison, Outcome, and Study Design) framework is used to systematically address or formulate clinical outcomes. Furthermore, GRADE (Grading of Recommendations, Assessment, Development, and Evaluations) is generally used to assess the quality of evidence in systematic reviews. Including these frameworks would improve the quality of the analysis. However, this analysis did not incorporate these aspects. The metadata from the narrated text of clinical trials will need to be curated differently to perform these analyses, which we plan to address in future studies.

## 4. Materials and Methods

### 4.1. Source for NR Gene Names and Safety Information

The gene names of NRs and associated pharmacology and toxicology information were sourced from various platforms, including the IUPHAR/BPS guide to pharmacology (https://www.guidetopharmacology.org, accessed on 6 May 2024), PubMed (https://pubmed.ncbi.nlm.nih.gov, accessed on 6 May 2024), Google Scholar (https://scholar.google.com, accessed on 6 May 2024), Pharmapendium (https://www.pharmapendium.com, accessed on 29 March 2024), and OFF-X (https://targetsafety.info, accessed on 8 May 2024). Additionally, information on knockout mouse phenotypes, when available, was collected from the Mouse Genome Informatics site (https://www.informatics.jax.org, accessed on 6 May 2024). Data pertaining to reported safety events for 44 out of the 48 human-expressed NRs were sourced from OFF-X (Clarivate). This translational safety intelligence portal aggregates information from diverse sources such as approval packages of regulatory agencies the FDA, European Medicines Agency (EMA), and Pharmaceuticals and Medical Devices Agency (PMDA), from the biomedical literature, congresses, scientific conferences, major clinical trial registries, real-world evidence studies, and pharmacovigilance databases (e.g., FDA Adverse Reporting System (FAERS), Japanese Adverse Drug Event Report (JADER)), as well as insights from key opinion leaders. As of March 2024, this database contains information on 15,608 protein targets (>75% human-expressed proteome). These targets are linked to over 2.5 million safety alerts with 13,400 unique commonly described adverse event terms for 37,105 drug molecules across various modalities. This portal served as the basis for obtaining safety alerts associated with 44 NRs for statistical analysis using JMP 16 (SAS Institute, Cary, NC, USA) and visualization via Tibco Spotfire (TIBCO, Palo Alto, CA, USA).

### 4.2. Safety Alerts and Gene Expression Data for 44 NRs

We obtained 92,858 safety alerts that connect 435 small molecule compounds in various stages of development to 44 NRs, spanning 27 unique organs. These alerts were sourced from peer-reviewed journals (44,961), regulatory agency communications (32,331), congresses/conferences (7960), clinical trial registries (5601), and company communications (1349) using OFF-X. The human RNA sequencing raw data were obtained from the Genotype-Tissue Expression (GTEx) project [32]. The RNA sequencing data for rats (GSE219045_Rat_FPKM_table_Mar22.xlsx), mice (GSE219045_Mouse_FPKM_table_Mar22.xlsx), dogs (GSE219045_Dog_FPKM_table_Jun22.xlsx), and cynomolgus monkeys (GSE219045_Cyno_FPKM_table_Mar22.xlsx) were acquired from the Gene Expression Omnibus (GEO) [101] and integrated within Omicsoft. Gene expression analysis of all 48 NRs, including 44 with safety alerts, was conducted utilizing these RNA-Seq data within the Omicsoft tool (Qiagen, Redwood City, CA, USA). Heatmaps were generated using the RNASeq quantification module with default settings in Omicsoft version 12.5.0.23 (Qiagen, Redwood City, CA, USA). All the heatmaps in this work were generated using gene FPKM (Fragments Per Kilobase per Million Mapped reads) values for the NR genes within Omicsoft. FPKM values were transformed to log_2_(FPKM + 0.1) to account for zero read values. The default RobustCenterScale normalization was used, where the median is subtracted and then divided by the median absolute deviation.

### 4.3. NR Gene Enrichment Analysis across Tissues

To determine gene enrichment in a specific tissue, we employed a method that utilizes the median expression of the target gene across all tissues and the mean expression of the target gene within individual tissues. First, we calculated the mean expression level of the target gene in the specific tissue of interest. Next, we determined the median expression level of the target gene across all tissues. To assess enrichment, we computed the ratio of the mean expression in the specific tissue to the median expression across all tissues. This enrichment ratio is given by the following formula: Enrichment Ratio = (Mean Expression in Specific Tissue)/(Median Expression Across All Tissues). If this ratio exceeds 1.2 (an arbitrary cut-off we set), the target gene is considered to be enriched in that tissue. This approach allows us to identify human tissues where the NR genes are expressed at levels higher than the baseline expression observed across all tissues. Omicsoft (Qiagen, CA, USA) was used to process untreated RNA-seq expression data from humans [101], mice, rats, dogs, and cynomolgus monkeys [33]. The mean, median, and fold change calculations were performed in JMP 16 (SAS Institute, Cary, NC, USA).

## 5. Conclusions

In conclusion, ensuring the safety of pharmacologically targeted small molecules, especially during lead candidate drug discovery, requires a comprehensive approach integrating in vitro, in vivo, in litero, and in silico studies. High failure rates in drug discovery and clinical studies often result from interactions with unintended target proteins, notably NRs, emphasizing the critical need for robust safety AI/ML-based in silico and early in vitro screening. This study identified a significant gap in understanding the safety consequences of small molecule interactions with various NRs; it also provided the nonclinical and clinical safety effects associated to functional interactions with 44 of 48 human-expressed NRs through the systematic analysis of various sources. Integrating NR-mediated outcomes dispersed across various sources into a unified framework for effective safety assessment is essential. Furthermore, some pharmaceutical and biotechnology companies often withhold institutional knowledge and preclinical data on discontinued drugs, leading to data gaps. Therefore, ongoing updates to our study and analysis are necessary as new information emerges for effective nonclinical safety assessment. Nonetheless, this study provided an overview of a significant number of NR family targets, their toxicological implications, mRNA expression in various species, and insights into different NR classes using currently available information. This compilation of NR-related toxicological effects and expression profiles may guide future drug development and enhance the safety assessment of candidate NR-interacting drugs and, whether intentional or unintentional, may ultimately increase the probability of success for new drug candidates in various therapeutic areas.

## Figures and Tables

**Table 1:** Total number of FDA-approved drugs for key nuclear receptor targets.

Nuclear Receptor Target	Number of FDA-Approved Drugs
GR	51
AR	25
PGR	20
ER	19
PPARa	12
VDR	12
RAR	9
MCR	8
PPARg	8
FXR	5
THRA	2
PPARd	1
SHP	1

**Table 2:** A comprehensive comparison of nuclear receptor (NR) targets in screening panels among four pharmaceutical companies and the FDA.

Target—Action	Novartis	AstraZeneca	FDA	AbbVie	Roche	Target Consensus
AR—Agonists	0	1	1	1	1	4
AR—Antagonists	0	1	1	1	1	4
GR—Agonists	0	1	0	1	1	3
PPARg—Agonists	0	0	1	1	1	3
ER—Agonists	0	0	1	0	1	2

## Data Availability

Where not included in the main body or as supplemental materials, the data that support the findings of this article may be made available on request from the corresponding author, [Mohan Rao]. Select data are not publicly available due to restrictions, such as information that could contain or compromise select intellectual property.

## Supplementary Materials

The following supporting information can be downloaded at: https://www.mdpi.com/article/10.3390/ph17070875/s1. Supplemental Table S1: This table compiles the reported adverse events associated with 44 nuclear receptors. It includes the target name, gene name, adverse event name, level of evidence, organ system affected, alert phase (discovery, preclinical, or clinical), species, and references. Supplemental Table S2: This table shows the mean, median, and relative fold change (FC) expression of these 38 nuclear receptors in various human tissues. Supplemental Table S3: This table compiles targets from five panels of Novartis, AstraZeneca, FDA, AbbVie, and Roche, as well as the consensus from these panels. Supplemental Figure S1: Heatmap of mouse mRNA expression of NRs. The color gradient from red to blue indicates high to low expression levels. The X-axis represents the NR name, and the Y-axis shows the tissues where the expression is noted. Supplemental Figure S2: Heatmap of dog mRNA expression of NRs. The color gradient from red to blue indicates high to low expression levels. The X-axis represents the NR name, and the Y-axis shows the tissues where the expression is noted. Supplemental Figure S3: Heatmap of cynomolgus monkey mRNA expression of NRs. The color gradient from red to blue indicates high to low expression levels. The X-axis represents the NR name, and the Y-axis shows the tissues where the expression is noted.
