# Supplementary material for: Safety Implications of Modulating Nuclear Receptors: A Comprehensive Analysis from Non-Clinical and Clinical Perspectives
Source: Pharmaceuticals (Basel). 2024 Jul 3;17(7):875. doi: 10.3390/ph17070875 (PMC11279859; doi:10.3390/ph17070875)
Supplement: Supplementary file 1 [file pharmaceuticals-17-00875-s001.zip › Figures_Modified_Supplemental.pptx]

## Slide 1
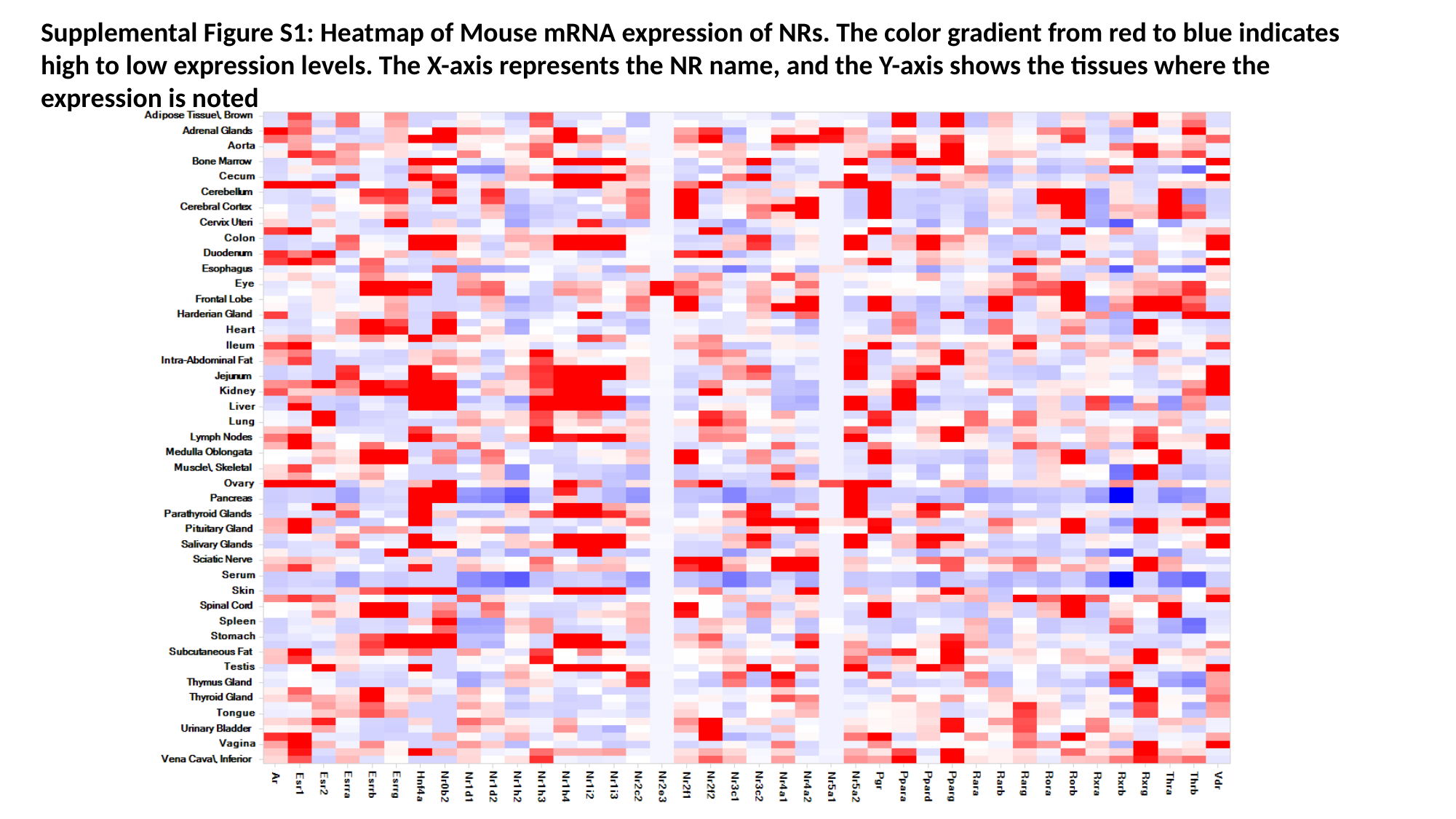

Supplemental Figure S1: Heatmap of Mouse mRNA expression of NRs. The color gradient from red to blue indicates high to low expression levels. The X-axis represents the NR name, and the Y-axis shows the tissues where the expression is noted

## Slide 2
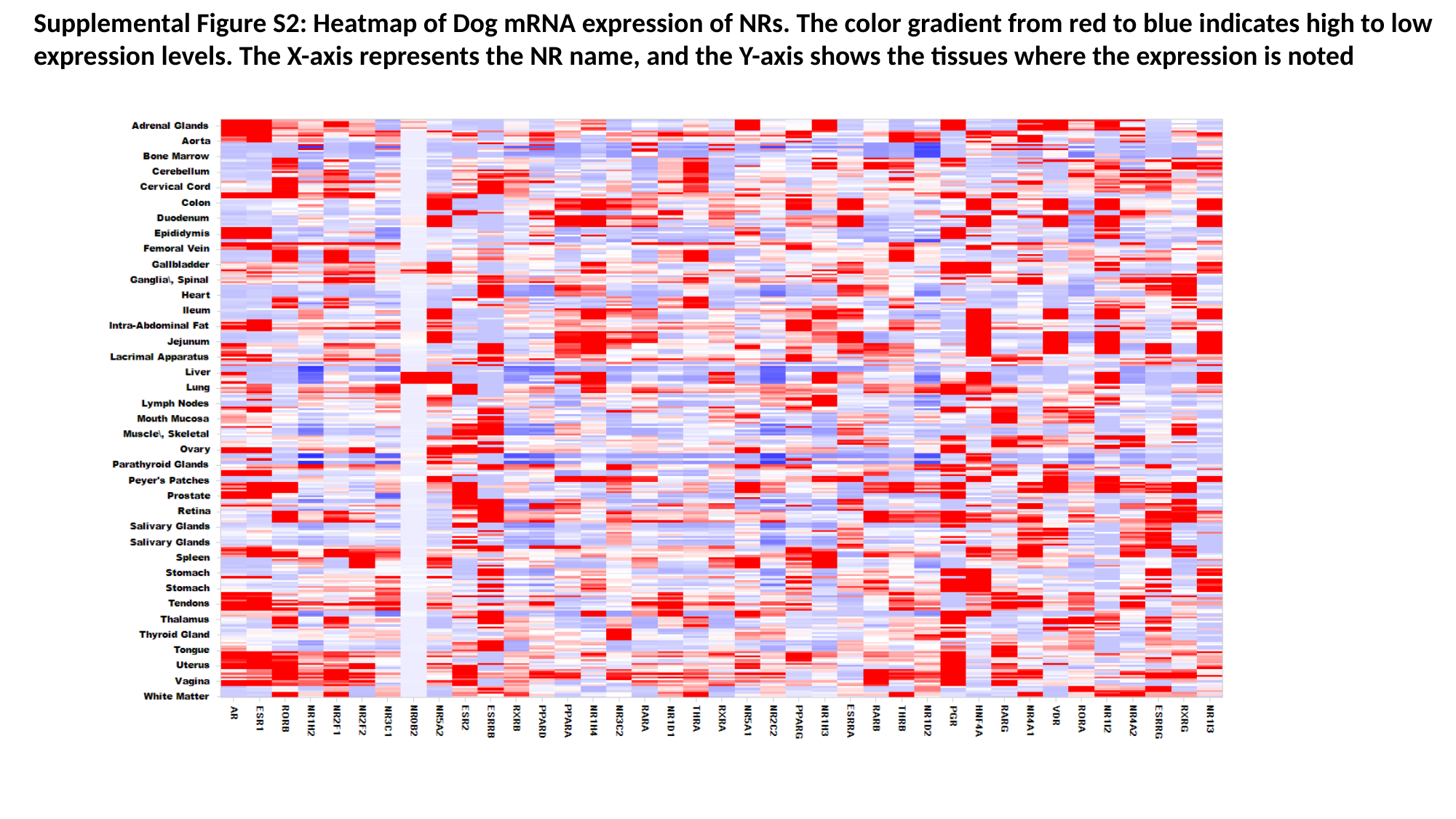

Supplemental Figure S2: Heatmap of Dog mRNA expression of NRs. The color gradient from red to blue indicates high to low expression levels. The X-axis represents the NR name, and the Y-axis shows the tissues where the expression is noted

## Slide 3
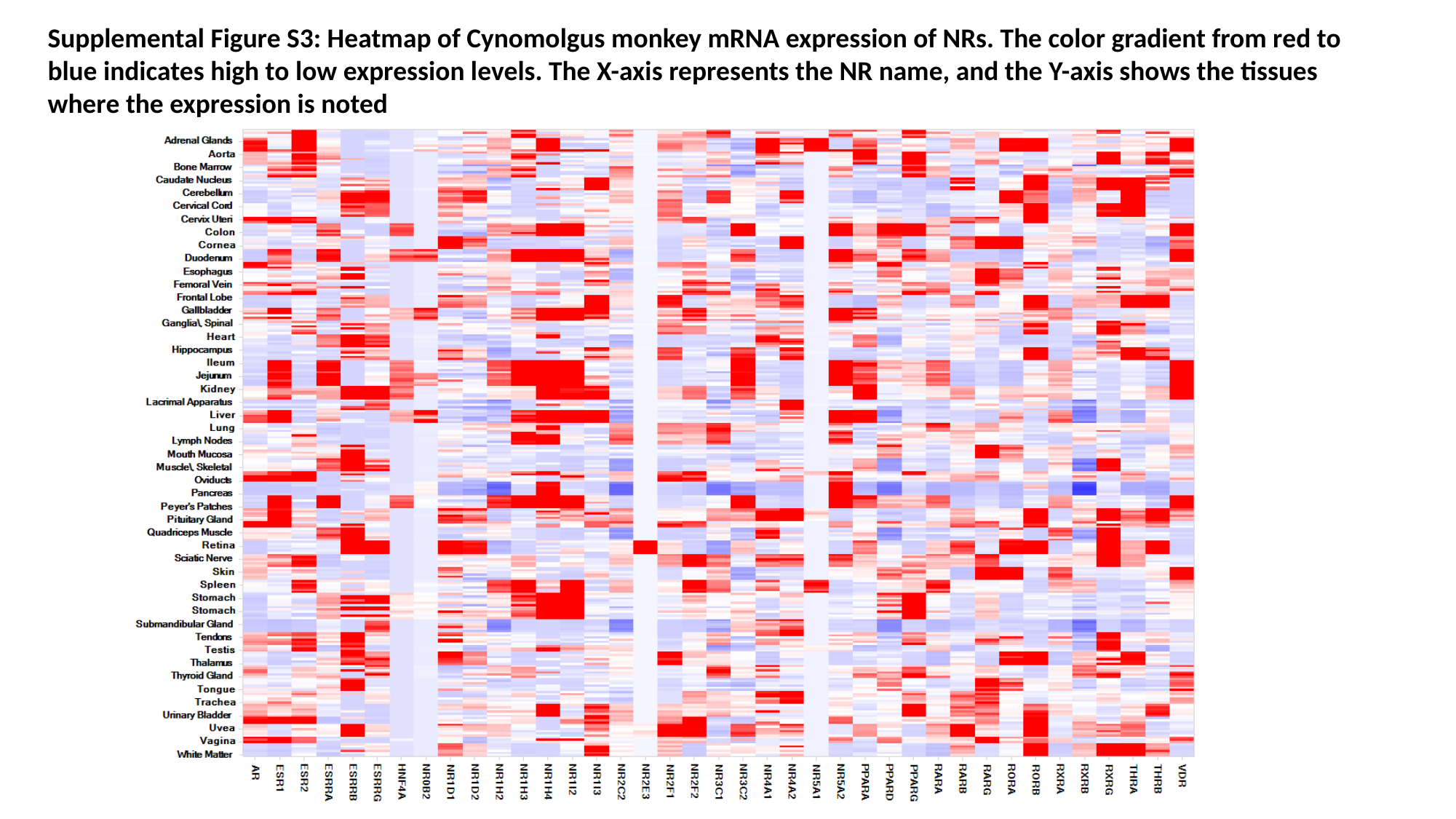

Supplemental Figure S3: Heatmap of Cynomolgus monkey mRNA expression of NRs. The color gradient from red to blue indicates high to low expression levels. The X-axis represents the NR name, and the Y-axis shows the tissues where the expression is noted
